# Retrospective Observational Study of Complete Blood Count (CBC) Parameters and ICU Mortality of COVID-19 Disease in Delta Variant and Omicron Variant in a Community-Based Hospital in New York City

**DOI:** 10.7759/cureus.34894

**Published:** 2023-02-12

**Authors:** Ruchi Yadav, Vivek Yadav, Sindhu Pokhriyal, Umar Zahid, Anjula Gandhi

**Affiliations:** 1 Internal Medicine, Brookdale University Hospital Medical Center, Brooklyn, USA; 2 Pulmonary and Critical Care, State University of New York Downstate Health Sciences University, New York, USA; 3 Internal Medicine, Interfaith Medical Center, Brooklyn, USA

**Keywords:** icu mortality, cbc parameters, omicron variant, delta variant, covid 19

## Abstract

Background: Severe Acute Respiratory Syndrome-Coronavirus-2 (SARS-CoV-2) is the official name of COVID-19, a respiratory infection that had the first case reported from the Hubei province of China on December 8, 2019. This virus is the main etiological agent behind the most dreaded pandemic of pneumonia that has spread to the entire world in a brief period and continues to pose a threat. The first wave corresponded with the period from February 2020 to June 2020, the Delta variant occurred around the middle of June 2021 and the Omicron wave was reported from December 2021 to February 2022.

Objective*:* This study aims to compare the Delta and the Omicron variants of COVID-19 infection in a community-based hospital in New York City considering the comparison of ICU admissions in both variants. We aim to study the comparison of complete blood count (CBC) parameters and inflammatory markers of patients admitted to ICU stratified by two waves of COVID-19 infection. We aim to analyze the association of CBC parameters at admission and the discharge during ICU stay in both variants. We also aim to study the association of CBC parameters at admission and discharge with ICU mortality in both variants.

Methods: We conducted a retrospective observational study based on data from randomly selected hospitalized patients with COVID-19 in a community-based hospital in New York City during the Delta variant and the Omicron wave. A total of 211 patients COVID-19 positive from June to July 2021 (Delta variant) and 148 patients from December to February 2022 (Omicron wave) were included in the study. A comparison was done between the basic characteristics of patients with and without ICU admissions in both variants of COVID-19. We compared the relationship of different parameters of CBC (hemoglobin (Hgb), white blood count (WBC), lymphocytes, neutrophils, and platelets) on ICU admission and further analyzed any changes associated with ICU mortality. Logistic regression was performed to evaluate the relationship of different presenting CBCs on patients’ disposition to ICU.

Result: A total of 211 patients (106 female) in the Delta wave (2021 variant) and 148 patients (80 female) in the Omicron wave (2022 variant) with an average ages of 60.9 ±18.10 (Delta variant) and 63.2 ± 19.10 (Omicron variant) were included in this study. There were 45 patients (21.3%) in the Delta wave and 42 patients (28.4%) in the Omicron wave were admitted to ICU. The average length of hospital stay was seven days in the Delta wave and nine days in the Omicron wave. No significant association was found between presenting cell count and ICU admission (p>0.05). Significant associations were found between different cell counts on admission and discharge and death in Delta waves except Hgb and platelets on admission. However, in the Omicron variant, a significant association was found only between WBC on admission and discharge, and Hgb and neutrophil on discharge with death in the univariate model.

Conclusion: Comparative study of different clinical parameters between the Delta and the Omicron variants of COVID-19 with the correlation of ICU stay and mortality can be used as a beneficial modality in assessing the outcome of the disease.

## Introduction

Wuhan city, the capital of Hubei province in China, became the center of the deadliest outbreak of pneumonia in December 2019. By January 7, 2020, a novel virus was isolated by Chinese scientists from the infected patients and was named Severe Acute Respiratory Syndrome-Coronavirus-2 (SARS-CoV-2, previously called 2019-nCoV), later designated as COVID-19 by the WHO [1]. The WHO labeled this disease as a pandemic on March 11, 2020 and, since then, the virus has infected more than 101 million individuals and killed over one million persons in the United States [2]. The Centers for Disease Control and Prevention (CDC) has been monitoring all variants and subvariants of the original SARS-CoV-2 virus circulating in the United States. SARS-CoV-2 underwent several mutations since the beginning and the mutants were associated with higher transmissibility, higher mortality, and hospitalization rates, which were named the variants of concern. B.1.617.2, better known as the "Delta" variant, which was initially discovered in India, overtook all other variants in the United States in the late summer/fall of 2021 [3]. The Omicron variant, B.1.1.529, was first detected in Botswana and South Africa and by December 2021, and it spread to the rest of all other continents with San Francisco identifying the first cases in United States [4]. In contrast to the first wave, where knowledge about COVID-19 was limited, the second and third waves saw a better medical response from healthcare systems, owing to a better scientific understanding of the epidemiology as well as the pathophysiology of the disease. The voluminous scientific data that was used to identify mortality risk factors and the efficacy of respiratory support measures have helped in curtailing the morbidity and mortality seen during the different waves of this pandemic [5]. Studies have shown the association of COVID-19 infection and severity with several hematological parameters such as platelets, white blood count (WBC), lymphocytes, neutrophils (together with neutrophil-lymphocyte and platelet-lymphocyte ratio), and hemoglobin (Hgb) [6]. The purpose of our retrospective study was to evaluate the index of ICU admissions in the Delta and the Omicron variants. We aimed to evaluate the association between specific CBC parameters at admission and discharge in both waves and their association with ICU mortality. Our study could be useful in the identification of effective biomarkers of this dreaded progressive disease and might be helpful for diagnosis, prevention of complications, and effective therapy of COVID-19 infection.

## Materials and methods

We conducted a retrospective observational study based on data from randomly selected hospitalized patients with COVID-19 infection in a community-based hospital in New York City during the Delta variant and the Omicron wave. A total of 211 patients COVID-19 positive from June 1, 2021, to July 31, 2021 (Delta variant) and 148 patients from December 15, 2021, to February 15,2022 (Omicron wave) were included in the study. Institutional Review Board (IRB) approved the study. All patients had tested positive for SARS-CoV-2 using a reverse transcriptase-polymerase chain reaction (RT-PCR) of the nasopharyngeal or oropharyngeal swab. COVID-19 variants were decided based on the timeline of the events with Delta variant common in 2021 and Omicron wave hitting the nation in 2022.

Data were presented as mean and standard deviation for continuous variables and categorical variables, frequencies, and proportions were generated. The mean of continuous variables was compared using independent t-tests or the Wilcoxon rank sum test where applicable. Categorical variables were compared using a chi-square or Fischer exact test. Basics characteristics and laboratory data were compared by the status of ICU admission among two waves of COVID-19 separately. We compared the relationship of different parameters of CBC (Hgb, WBC, lymphocytes, neutrophils, and platelets) on ICU admission and further compared them between two waves of COVID-19. Logistic regression was performed to evaluate the relationship of different presenting CBCs on patients’ disposition to ICU. The different blood counts on admission and discharge were also entered into logistic regression models to evaluate the association between these cell counts and death. Both crude and multivariable models (adjusted for age, sex, race, BMI, and co-morbidities (hypertension, diabetes, coronary artery disease (CAD), cancer, end-stage renal disease (ESRD), chronic obstructive pulmonary disease (COPD)/asthma, smoking, and mechanical ventilation status)) were run. Separate regression models were run for each variable of blood as of strong correlation among them. All statistical tests were two-sided with a significant level of 0.05. Statistical analyses were performed using Statistical Software STATA 14.2.

## Results

Table 1 compares the basic clinical and demographic characteristics of the study participants stratified by the disposition to ICU in the Delta and the Omicron variants. A total of 211 patients (106 female) in the Delta wave (June-July 2021 variant) and 148 patients (80 female) in the Omicron wave with an average age of 60.9 ±18.10 (Delta variant) and 63.2 ± 19.10 (Omicron variant) were included in this study. 36.5% and 74.3% of patients were African American in the Delta and the Omicron waves, respectively. There were 45 (21.3%) in the Delta wave and 42 patients (28.4%) in the Omicron wave that were admitted to ICU. Among the patients admitted to ICU, 73% had hypertension and 49% had diabetes in the Delta wave while 69% had hypertension and 62% diabetes in the Omicron wave. 26 patients (12.32%) in the Delta variant and 23 (15.54%) in the Omicron wave died regardless of ICU stay. The average length of hospital stay was seven days in the Delta wave and nine days in the Omicron wave.

**Table 1:** Basic characteristics of patients enrolled in the study stratified by ICU admission IQR: Interquartile range; CAD: Coronary artery disease; COPD: Chronic obstructive pulmonary disease; ESRD: End-stage renal disease; NSAID: Non-steroidal anti-inflammatory drugs; ACE-I: Angiotensin converting enzyme inhibitor; WBC: White blood count; CRP: C-reactive protein; LDH: Lactate dehydrogenase; Hgb: Hemoglobin; SE: Standard error

	Delta variant				Omicron variant			
Variables	All Patients (N=211)	No ICU (N=166, 78.7%)	ICU (N=45, 21.3%)	p-value	All Patients (N=148)	No ICU (N=106, 71.6%)	ICU (N=42, 28.4%)	p-value
Age, mean (SD)	60.9 (18.1)	59.7 (18.3)	65.0 (16.3)	0.08	63.2 (19.1)	62.7 (20.2)	64.3 (16.3)	0.65
BMI, median (IQR)	28.3 (24.6, 34.1)	28.3 (24.8, 34.0)	28.5 (23.6, 32.9)	0.65	27.5 (22.6, 33.3)	26.7 (22.5, 33)	30.3 (23.5, 33.9)	0.43
Gender, n (%)								
Male	105 (49.76)	91 (85.85)	15 (14.15)	0.86	68 (45.95)	55 (80.88)	13 (19.12)	0.26
Female	106 (50.24)	91 (86.67)	14 (13.33)		80 (54.05)	70 (87.50)	10 (12.50)	
Race, n (%)								
African American	77 (36.49)	59 (35.5)	18 (40.0)	0.75	110 (74.32)	76 (72.38)	34 (80.95)	0.68
Caucasian	6 (2.84)	6 (3.61)	0 (0.00)		6 (4.05)	4 (3.81)	2 (4.76)	
Hispanic	39 (18.48)	29 (17.5)	10 (22.2)		14 (9.46)	12 (11.43)	2 (4.76)	
Non-Hispanic	61 (28.91)	49 (29.5)	12 (26.7)		1 (0.68)	1 (0.96)	0 (0.00)	
Other	28 (19.23)	23 (13.9)	5 (11.1)		17 (11.49)	12 (11.43)	4 (9.52)	
Co-Morbidities, n (%)								
Hypertension	129 (61.14)	96 (57.83)	33 (73.33)	0.06	95 (64.19)	66 (62.26)	29 (69.05)	0.44
Diabetes Mellitus	81 (38.39)	59 (35.54)	22 (48.89)	0.11	69 (46.62)	43 (40.57)	26 (61.90)	0.02
CAD	32 (15.17)	23 (13.86)	9 (20)	0.31	27 (18.24)	17 (16.06)	10 (23.81)	0.27
COPD	26 (12.32)	17 (10.24)	9 (20.0)	0.07	24 (16.22)	18 916.98)	6 (14.29)	0.68
Cancer	19 (9.00)	14 (8.43)	5 (11.11)	0.57	11 (7.43)	10 (9.43)	1 (2.38)	0.18
ESRD	12 95.69)	8 (4.82)	4 (8.89)	0.29	11 (7.43)	6 (5.66)	5 (11.90)	0.19
Smoking	78 (36.97)	62 9 (37.35)	16 (35.56)	0.82	57 (38.51)	42 (39.62)	15 (35.71)	0.66
NSAID	14 (6.64)	12 (7.23)	2 (4.44)	0.74	16 (10.81)	10 (9.43)	6 (14.29)	0.39
ACE-I	62 (29.38)	45 (27.11)	17 (37.78)	0.16	54 (36.49)	41 (38.68)	13 (30.95)	0.38
Statin	97 (45.97)	69 (41.57)	28 (62.22)	0.014	65 (43.92)	47 (44.34)	18 (42.86)	0.87
Mean arterial pressure ≥ 65 <65	193 (91.47) 18 (8.53)	155 (93.37) 11 (6.63)	38 (84.44) 7 (15.56)	0.05	133 (89.86) 15 (10.14)	97 (91.51) 9 (8.49)	36 (85.71) 6 (14.29)	0.29
Labs, mean (SD)								
Hgb on admission	12.7 (6.4)	12.28(2.5)	14.07 (11.5)	0.11	11.9 (2.44)	12.08 (2.41)	11.71 (2.51)	0.39
Hgb on discharge	11.5 2.18	11.6 (2.10)	11.21 (2.44)	0.34	10.9 (2.39)	11.17 (2.3)	10.40 (2.4)	0.08
WBC on admission	7.7 (4.22)	7.71 (4.42)	8.16 (4.02)	0.52	8.84 (5.72)	9.18 (5.12)	7.99 (4.01)	0.26
WBC on discharge	9.7 (7.2)	9.56 (5.54)	11.27 (5.89)	0.189	8.8 (6.21)	8.73 (4.71)	9.33 (5.21)	0.59
Neutrophil on admission	5.82 (3.88)	5.66 (2.91)	6.42 (3.77)	0.27	6.74 (5.51)	7.11 (4.90)	5.81 (3.55)	0.19
Neutrophil on discharge	6.13 (3.59)	5.91 (3.56)	6.94 (3.62)	0.12	5.92 (3.89)	5.77 (3.31)	6.30 (4.56)	0.47
Platelets on admission	242 (113)	248 (115)	220 (103)	0.15	243 (109)	244 (102)	242 (114)	0.92
Lymphocytes on admission	1.25 (0.90)	1.23 (0.74)	1.30 (0.89)	0.63	1.31 (0.95)	1.28 (0.97)	1.41 (0.91)	0.43
Lymphocyte on discharge	1.57 (1.02)	1.63 (1.07)	1.40 (0.78)	0.22	1.63 (1.21)	1.73 (1.03)	1.40 (1.01)	0.15
D-dimer, mean (SE)	2804 (1878)	2064 (467)	5247 (1607)	0.01	3945 (3045)	2593 (987)	6611 (1495)	0.02
CRP, mean (SE)	11.4 (7.92)	10.7 (2.3)	13.9 (1.5)	0.45	10.3 (8.4)	8.6 (0.95)	13.4 (1.57)	0.007
Lactate, mean (SE)	2.7 (2.5)	2.1 (0.13)	4.4 (0.58)	<0.001	3.6 (4.5)	2.6 (0.28)	5.44 (0.98)	0.009
LDH, mean (SE)	1148 (845)	985 (49.5)	1697 (198.9)	<0.0001	1083 (877)	843 (50.7)	1587 (202.8)	<0.0001
Ferritin, mean (SE)	707 (562)	568 (64.8)	1159 (216.4)	0.005	729 (650)	468 (60.2)	1287 (140)	<0.0001
Length of stay, median (IQR)	7 (4, 14)	6 (3, 12)	11 (6, 29)	<0.001	9 (4, 18)	7 (3, 14)	15 (9, 30)	<0.001
Require mechanical ventilation								
Yes	24 (11.37)	3 (1.81)	21 (46.67)	<0.001	24 (16.22)	1 (0.94)	23 (54.76)	<0.001
No	187 (88.63)	163 (98.19)	24 (53.33)		124 (83.78)	105 (99.06)	19 (45.24)	
Disposition								
Discharged	185 (87.68)	152 (91.57)	33 (73.33)	0.001	125 (84.46)	93 (87.74)	32 (16.19)	0.080
Deceased	26 (12.32)	14 (8.43)	12 (26.67)		23 (15.54)	13 (12.26)	10 (23.81)	

The relationship between different presenting cell counts and ICU admission is presented in Table 1. No significant association was found between presenting cell count and ICU admission (p>0.05). Further, different cell counts were compared among the participants admitted to ICU in two waves and no significant difference was found among these cell lines in two waves as shown in Table 2.

**Table 2:** Comparison of complete blood count (CBC) parameters and inflammatory markers of patients admitted to ICU stratified by two waves of COVID-19 WBC: White blood count; CRP: C-reactive protein; LDH: Lactate dehydrogenase; Hgb: Hemoglobin; SE: Standard error

Labs	Delta variant		Omicron variant		p-value
	All Patients	ICU	All Patients	ICU	
Hgb on admission	12.7 (6.4)	14.07 (11.5)	11.9 (2.44)	11.71 (2.51)	0.25
Hgb on discharge	11.5 2.18	11.21 (2.44)	10.9 (2.39)	10.40 (2.4)	0.12
WBC on admission	7.7 (4.22)	8.16 (4.02)	8.84 (5.72)	7.99 (4.01)	0.85
WBC on discharge	9.7 (7.2)	11.27 (5.89)	8.8 (6.21)	9.33 (5.21)	0.27
Neutrophil on admission	5.82 (3.88)	6.42 (3.77)	6.74 (5.51)	5.81 (3.55)	0.47
Neutrophil on discharge	6.13 (3.59)	6.94 (3.62)	5.92 (3.89)	6.30 (4.56)	0.52
Platelets on admission	242 (113)	220 (103)	243 (109)	242 (114)	0.39
Lymphocytes on admission	1.25 (0.90)	1.30 (0.89)	1.31 (0.95)	1.41 (0.91)	0.69
Lymphocyte on discharge	1.57 (1.02)	1.40 (0.78)	1.63 (1.21)	1.40 (1.01)	0.91
D-dimer, mean (SE)	2804 (1878)	5247 (1607)	3945 (3045)	6611 (1495)	0.543
CRP, mean (SE)	11.4 (7.92)	13.9 (1.5)	10.3 (8.4)	13.4 (1.57)	0.83
Lactate, mean (SE)	2.7 (2.5)	4.4 (0.58)	3.6 (4.5)	5.44 (0.98)	0.37
LDH, mean (SE)	1148 (845)	1697 (198.9)	1083 (877)	1587 (202.8)	0.69
Ferritin, mean (SE)	707 (562)	1159 (216.4)	729 (650)	1287 (140)	0.69

Regression analysis results showed that there was no significant association between different cell counts on admission and patients’ disposition to ICU as shown in Table 3. To further explore the influence of cell count on ICU admission we use the normal range of the WBC as the cutoff value but still, no association was found between WBC and ICU admission.

**Table 3:** The associations of different blood counts on admissions and disposition to ICU * Adjusted for age, sex, race, BMI, co-morbidities (hypertension, diabetes, CAD, cancer, ESRD, COPD/asthma, smoking), mean arterial blood pressure on presentation, and mechanical ventilation status. # Separate regression models were run for each variable as of strong correlation among them. WBC: White blood count; OR: Odds ratio; CI: Confidence interval; CAD: Coronary artery disease; ESRD: End-stage renal disease; COPD: Chronic obstructive pulmonary disease

Variables	Delta variant				Omicron variant			
	Crude Model		Adjusted model*		Crude Model		Adjusted model*	
	OR (95% CI)	p-value	OR (95% CI)	p-value	OR (95% CI)	p-value	OR (95% CI)	p-value
WBC on admission								
Continuous	1.03 (0.95, 1.15)	0.52	1.02 (0.93, 1.10)	0.72	0.96 (0.88, 1.03)	0.26	0.93 (0.84, 1.02)	0.11
Categories								
<10			Reference				Reference	
>=10	1.18 (0.55, 2.51)	0.66	1.22 (0.54, 2.3)	0.625	0.78 (0.55, 1.69)	0.53	0.68 (0.50, 1.44)	0.26
Hgb on admission	1.03 (0.97, 1.10)	0.23	1.06 (0.98, 1.13)	0.12	0.94 (0.81, 1.08)	0.41	0.88 (0.74, 1.04)	0.15
Lymphocyte on admission	1.09 (0.77, 1.53)	0.62	1.04 (0.77, 1.48)	0.85	1.15 (0.81, 1.65)	0.43	1.06 (0.72, 1.58)	0.76
Neutrophil on admission	1.05 (0.96, 1.14)	0.27	1.03 (0.94, 1.13)	0.45	0.94 (0.86, 1.02)	0.19	0.92 (0.83, 1.01)	0.08
Platelets on admission	0.99 (0.99, 1.01)	0.16	0.99 (0.98, 1.001)	0.061	0.99 (0.98, 1.003)	0.92	1.004 (0.99, 1.005)	0.84

The relationship of different blood cell counts (admission and discharge) and death is presented in Table 4. Significant associations were found between different cell counts on admission and discharge and death in the Delta wave except for Hgb and platelets on admission. However, in the Omicron variant, a significant association was found only between WBC on admission and discharge, and Hgb and neutrophil on discharge with death in the univariate model. The significance of these cell lines still exists after adjusting for covariables. In the adjusted regression model (separate regression analysis for each cell count), a significant association between odds of death and WBC on admission (odds ratio (OR) 1.12, p=0.019), WBC on discharge (OR 1.36, p<0.001), Hgb on discharge (OR 0.59, p<0.001), lymphocyte on admission (OR 0.29, p=0.018), lymphocyte on discharge (OR 0.29, p=0.007), neutrophil on admission (OR 1.18, p=0.003), and neutrophil on discharge (OR 1.32, p<0.001) were found in Jun-July variant (Delta). In the adjusted regression model of the Omicron variant significant association between death and WBC on discharge (OR 1.21, p=0.03), Hgb on discharge (OR 0.55, p<0.001), and neutrophil on discharge (OR 1.24, p=0.002) were found. WBC counts were further categorized at their normal upper limit to further explore the influence of WBC as a cutoff value to death. The regression analysis showed that there was a significant association of WBC on admission (OR 3.18, p=0.012) and discharge (OR 9.65, p<0.001) in the Delta variant when we use normal upper limit as the cutoff value (≥10.0 x 10^9^/L), while in Omicron variate WBC cutoff value was only significant for WBC on discharge and death (OR 4.43, OR=0.01) as shown in Table 4.

**Table 4:** The association of different blood counts (at admission and at discharge) and death * Adjusted for age, sex, race, BMI, co-morbidities (hypertension, diabetes, CAD, cancer, ESRD, COPD/asthma, smoking), mean arterial blood pressure on presentation, and mechanical ventilation status. # Separate regression models were run for each variable as of strong correlation among them. OR: Odds ratio, CI: Confidence interval, Hgb: Hemoglobin; CAD: Coronary artery disease; ESRD: End-stage renal disease; COPD: Chronic obstructive pulmonary disease

Variables	Delta variant				Omicron variant			
	Crude Model		Adjusted model*		Crude Model		Adjusted model*	
	OR (95% CI)	p-value	OR (95% CI)	p-value	OR (95% CI)	p-value	OR (95% CI)	p-value
WBC on admission								
Continuous	1.10 (1.01, 1.19)	0.019	1.12 (1.01, 1.23)	0.019	1.014 (0.94, 1.08)	0.73	1.01 (0.94, 1.09)	0.704
Categories								
<10			Reference				Reference	
≥10	2.65 (1.13, 6.23)	0.025	3.18 (1.25, 6.54)	0.021	2.18 (0.88, 5.38)	0.09	2.21 0.83, 5.84)	0.11
WBC on discharge								
Continues	1.21 (1.15, 1.32)	<0.001	1.36 (1.17, 1.57)	<0.001	1.17 (1.06, 1.29)	0.001	1.21 (1.06, 1.37)	0.03
Categories								
<10	Reference				Reference			
≥10	5.01 (2.01,12.54)	0.001	9.65 (3.13, 20.34)	<0.001	3.21 (1.29, 7.99)	0.012	4.43 (1.42, 11.6)	0.01
Hgb on admission	0.88 (0.74, 1.04)	0.15	0.83 (0.67, 1.01)	0.06	1.06 (0.88, 1.27)	0.52	1.03 (0.84, 1.27)	0.719
Hgb on discharge	0.63 (0.51, 0.78)	<0.001	0.59 (.45, 0.76)	<0.001	0.64 (0.51, 0.81)	<0.001	0.55 (0.404, 0.745)	<0.001
Lymphocyte on admission	0.284 (0.11, 0.74)	0.011	0.29 (0.102, 0.806)	0.018	0.64 (0.34, 1.21)	0.17	0.58 (0.304, 1.120	0.11
Lymphocyte on discharge	0.29 (0.13, 0.64)	0.002	0.29 (0.12, 0.71)	0.007	1.07 (0.77, 1.49)	0.67	1.05 (0.72, 1.52)	0.81
Neutrophil on admission	1.14 (1.04, 1.24)	0.03	1.18 (1.06, 1.32)	0.003	1.02 (0.95, 1.09)	0.51	1.01 (0.93, 1.08)	0.87
Neutrophil on discharge	1.21 (1.09, 1.35)	<0.001	1.32 (1.13, 1.53)	<0.001	1.18 (1.06, 1.32)	0.002	1.24 (1.08, 1.542)	0.002
Platelets on admission	0.99 (0.994, 1.003)	0.48	0.99 (0.992, 1.003)	0.61	0.99 (0.992, 1.002)	0.288	0.99 (0.99, 1.002)	0.309

## Discussion

Almost every patient admitted with COVID-19 infection undergoes a routine CBC, which provides crucial information that further affects clinical management during a hospital stay. According to a study done in five different nations throughout the world, CBC is the most widely used initial laboratory test in all patients hospitalized for various indications [7]. There are an increasing number of studies being done to highlight the different clinical characteristics of several variants of COVID-19 infection, and one of them is the prospective longitudinal observational study done by Cristina Menni et al quantifying the differences in symptom prevalence, risk of hospital admission, and symptom duration among the vaccinated population in Delta and Omicron variant [8]. Important hematological parameters in COVID-19 infection were studied by Regolo et al recently concluding that patients with a high neutrophil/lymphocyte ratio (NLR) at admission were more likely to have disease progression as well as ICU admission and mortality when compared to those with lower/normal NLR [9]. NLR represents a rapid, widely available, and inexpensive tool that could be useful in the management and early risk stratification of patients with COVID-19 [9]. Saurabh et al, in their retrospective analysis done in April-May 2021, studied the hematological parameters including Hgb, platelet count, total leukocyte count (TLC), neutrophils, lymphocytes, NLR, and absolute eosinophil count (AEC) in COVID-19 positive patients within 24 hours of admission concerning both ICU and non-ICU admissions [10]. They found that relative neutrophilia 70% (p-value 0.007), relative lymphopenia 20% (p-value 0.002), and NLR 9.1 (p-value 0.001) were all significantly associated with ICU disposition. However, there was no association between Hgb levels (p-value 0.29) or platelet counts (p-value 0.61) and COVID-19 patients’ admission to the ICU in their study. They concluded that routine hematological tests like CBC are quick, cost-effective predictors for patients with severe COVID-19, especially in limited resource settings [10]. In their analysis of 90 COVID-19-positive patients (51 ICU and 39 non-ICU), Pozdnyakova et al, who focused more on the WBC, discovered that all COVID-19 patients had striking quantitative and morphologic WBC changes with a significant positive correlation between disease severity, neutrophilia, and lymphopenia [11]. In our study in an adjusted regression model (separate regression analysis for each cell count), a significant association between odds of death and WBC on admission (OR 1.12, p=0.019), WBC on discharge (OR 1.36, p<0.001), Hgb on discharge (OR 0.59, p<0.001), lymphocyte on admission (OR 0.29, p=0.018), lymphocyte on discharge (OR 0.29, p=0.007), neutrophil on admission (OR 1.18, p=0.003), and neutrophil on discharge (OR 1.32, p<0.001) were found in the Delta variant. In the adjusted regression model of the Omicron variant, significant association between death and WBC on discharge (OR 1.21, p=0.03), Hgb on discharge (OR 0.55, p<0.001), and neutrophil on discharge (OR 1.24, p=0.002) were found. Although there have been many studies that have analyzed the CBC characteristics of different COVID waves, to our knowledge this is the first study that compared the CBC characteristics between the Delta and Omicron COVID waves at admission and discharge. Richardson et al, in their study on 5,700 patients hospitalized with COVID-19 infection in the NYC area, studied the presenting patient characteristics, comorbidities, and outcomes [12]. Hypertension, obesity, and diabetes were the most common comorbidities and among patients who were discharged or died, 14.2% were treated in the intensive care unit, 12.2% received invasive mechanical ventilation, 3.2% were treated with kidney replacement therapy, 21% died [12]. In our study group, 21.3% (delta variant) and 28.4% (omicron variant) of the total patients were admitted to ICU. Among the patients admitted to ICU, 73% had hypertension and 49% diabetes in Delta, while 69% had hypertension and 62% diabetes in Omicron wave. There have been studies showing a comparison of clinical characteristics between the Delta variant and the Omicron variant of SARS-COV-2 infections [13] however not much research has been done comparing the CBC parameters and emphasizing its relevance.

There are several limitations to our study. First, the number of patients were less in the Omicron variant as compared to the Delta variant. Second, the study population only included patients within the New York City area. Third, the data were collected from the electronic health record database precluding the minute details. Fourth, subgroup descriptive statistics were not adjusted for potential confounders. 

## Conclusions

The average length of hospital stay was seven days in the Delta wave and nine days in the Omicron wave in our hospital-based study. Regression analysis results showed that there was no significant association between different cell counts on admission and patients’ disposition to ICU in both variants of COVID-19. Significant associations were found between different cell counts on admission and discharge and death in the Delta wave except for Hgb and platelets on admission. However, in the Omicron variant, a significant association was found only between WBC on admission and discharge and Hgb and neutrophil on discharge with death in the univariate model. More studies need to be conducted in the future to emphasize the importance of the clinical parameters in standardizing the treatment options for COVID-19 variants.
